# Resistance of neonatal endotracheal tubes: a comparison of four commercially available types

**DOI:** 10.1186/cc12098

**Published:** 2013-03-19

**Authors:** J Spaeth, D Steinmann, J Guttmann, S Schumann

**Affiliations:** 1 University Medical Center Freiburg, Germany

## Introduction

In mechanically ventilated neonates the flow-dependent resistance of the endotracheal tube (ETT) causes a noticeable pressure difference between airway and tracheal pressure [1]. This may potentially lead to retardation of the passive driven expiration and dynamic lung inflation consecutively but more importantly increases the work of breathing. The aim of this study was to compare the resistive pressure drop of four commercially available neonatal ETTs with internal diameter (ID) of 2.0 mm.

## Methods

The pressure-flow relationship of neonatal ETTs (internal diameter 2.0 mm) of four different manufacturers (Mallinckrodt, Hi-Contour, Covidien, Dublin, Ireland; Portex, Cole's Neonatal Tube, Smith Medical, St Paul, MN, USA; Rusch, Silko Clear, Teleflex Medical, Kernen, Germany; and Vygon, Pediatric Endotracheal Tube, Ecouen, France) was determined in a physical model consisting of a tube connector, an anatomically curved ETT and an artificial trachea. The model was ventilated with a sinusoidal gas flow with an amplitude of ±100 ml/ second and a ventilation rate ranging from 40 to 60 cycles/minute. The coefficients of an approximation equation considering ETT resistance were fitted separately to the measured pressure-flow curves for inspiration and expiration.

## Results

The pressure drop profiles of all ETTs were nonlinearly flow dependent. The expiratory pressure drop (P_exp_) slightly exceeded the inspiratory one (P_insp_). The ETT of Portex Cole's Neonatal Tube had a significant lower pressure drop (P_insp _8.8 ± 0.3 cmH_2_O vs. P_exp _-11.2 ± 0.2 cmH_2_O at an air flow of 100 ml/second) compared with all other ETTs (Mallinckrodt: P_insp _19.2 ± 0.4 cmH_2_O vs. P_exp _-24.3 ± 0.7 cmH_2_O; Rüsch: P_insp _28.3 ± 0.8 cmH_2_O vs. P_exp _-33.0 ± 0.8 cmH_2_O; Vygon: P_insp _19.2 ± 0.4 cmH_2_O vs. P_exp _-22.6 ± 0.6 cmH_2_O, all *P <*0.05) for all flow and ventilation rates, respectively.

## Conclusion

The ETT resistance highly contributes to the total airway resistance in neonatal ventilation. The flow-dependent pressure drop of shouldered Cole's Neonatal Tube (Portex) was up to 70% less in inspiration (resp. 67% in expiration) compared with straight tubes with an internal diameter corresponding to the narrow part of Cole's tube. We conclude that neonatal intubation with a Cole's tube can clearly reduce the resistive load due to the endotracheal tube and thus potentially prevent additionally work of breathing.
